# Dietary Cholesterol, Lipid Levels, and Cardiovascular Risk among Adults with Diabetes or Impaired Fasting Glucose in the Framingham Offspring Study

**DOI:** 10.3390/nu10060770

**Published:** 2018-06-14

**Authors:** Hsuan-Ping Lin, Siyouneh Baghdasarian, Martha R. Singer, Melanie M. Mott, M. Loring Bradlee, Richard T. Pickering, Lynn L. Moore

**Affiliations:** Section of Preventive Medicine and Epidemiology, Department of Medicine, Boston University School of Medicine, Boston, MA 02118, USA; hpl1201@bu.edu (H.-P.L.); siyob@bu.edu (S.B.); msinger@bu.edu (M.R.S.); melaniemmott@yahoo.com (M.M.M.); lbradlee@bu.edu (M.L.B.); rtpicker@bu.edu (R.T.P.)

**Keywords:** dietary cholesterol, type 2 diabetes, impaired fasting glucose, Dietary Guidelines, lipid levels, cardiovascular disease, eggs

## Abstract

Previous recommendations to limit dietary cholesterol intake have been eliminated for most adults. Questions remain about whether dietary cholesterol has adverse cardiovascular effects among individuals with impaired fasting glucose or diabetes (IFG/T2DM). We used data for 993 adults (40.9% female), ages 35–<65 years, with prevalent IFG/T2DM in the prospective Framingham Offspring Study to address this question. Dietary cholesterol was assessed using 3-day diet records at exams 3 and 5 and used to classify subjects into sex-specific tertiles of mean cholesterol intake. Outcomes included fasting lipid levels over 20 years and incident cardiovascular disease (CVD). Statistical analyses included repeated measures mixed regression models and Cox proportional hazards models to adjust for confounding. Among adults with T2DM/IFG, there was no consistent association between dietary cholesterol intake and fasting low-density lipoprotein (LDL), high-density lipoprotein (HDL), LDL/HDL ratio, or triglycerides over 20 years of follow-up. In longitudinal analyses, the adjusted hazard ratio for CVD in the highest (vs. lowest) sex-specific tertile of cholesterol intake was 0.61 (95% CI: 0.41, 0.90). These analyses provide no evidence of an adverse association between dietary cholesterol and serum lipid levels or atherosclerotic CVD risk among adults with prevalent IFG/T2DM.

## 1. Introduction

An estimated 30.3 million people in the U.S (9.4% of the population) have type 1 or type 2 diabetes which are known to be associated with a number of cardiovascular disease (CVD) risk factors and other complications [1]. An additional 33.9% of adults aged 18 years or older have prediabetes, a finding that is also associated with increased CVD risk [1,2]. Identification of modifiable factors for CVD among this group of high-risk adults is a high priority.

Early *Dietary Guidelines for Americans* for many years recommended limiting dietary cholesterol intake to no more than 300 mg per day [3]. However, a number of longitudinal studies and interventional trials failed to support the earlier presumed association between dietary cholesterol and CVD [4,5,6]. As a result, the report from the US Department of Agriculture (USDA)’s 2015 *Dietary Guidelines for Americans* stated that “cholesterol is no longer considered a nutrient of concern for overconsumption” among healthy adults [7]. In fact, some studies have shown that higher dietary cholesterol intake is associated with beneficial changes in lipid profiles including increased numbers of large low-density lipoprotein (LDL) and high-density lipoprotein (HDL) particles, decreased numbers of small dense LDL particles, and no adverse association with the LDL/HDL ratio [8,9,10,11,12,13].

Questions remain about the relation between dietary cholesterol intake and CVD risk among individuals with diabetes and pre-diabetes. Several epidemiology studies, including two systemic reviews and a meta-analysis, found that higher egg intake (as a marker of dietary cholesterol) was associated with increased CVD risk amongst people with diabetes [14,15], while other studies have not shown such an association [16]. Some analyses examining the effects of higher intakes of overall dietary cholesterol have suggested an association with increased CVD risk among adults with prevalent diabetes [17], although this evidence is far from conclusive.

For the following analyses, we used data from the prospective Framingham Offspring Study (FOS) to examine the association between dietary cholesterol and change in lipid levels over time as well as the risk of incident CVD among individuals with prevalent diabetes.

## 2. Materials and Methods

### 2.1. Study Population

This study used data from the Framingham Offspring Study (FOS). Details of this study have been previously described [18]. Enrollment began in 1972 with 5124 participants who were offspring (and spouses) of the original Framingham Heart Study subjects. Data related to physical/medical status, laboratory values, and lifestyle factors were collected at exam visits that occurred at approximately four-year intervals.

For the current prospective analyses, we have included participants at exam visits 3, 4, or 5 (when dietary data were available), starting with at the first exam visit at which they were 35–<65 years of age (n = 4064). We excluded 300 subjects who had extremes of dietary intake, defined as follows: (1) average daily energy intake <1200 or >4000 kilocalories in men, or <1000 or >3500 kilocalories in women; (2) average daily percent of energy from alcohol >20%; (3) average weekly intake of >56 ounce-equivalents of meat, or >56 ounce-equivalents of poultry, or >56 ounce-equivalents of fish (but not total meat, poultry, and fish); (4) >35 eggs per week; or (5) >100 ounce-equivalents of soy, nuts, seeds, and legumes. We also excluded 1006 who did not provide food diaries and 1403 who had no prevalent impaired fasting glucose (IFG) or Type 2 diabetes mellitus (T2DM) at or before exam visit 5, as well as 204 with missing lipid data, and 68 with missing data on confounding variables of interest. This yielded a final analytic sample of 993 subjects with prevalent T2DM or impaired fasting glucose for the analyses examining dietary cholesterol intake and lipid levels through exam visit 8. Finally, 82 subjects with prevalent CVD at baseline were excluded for the analyses related to dietary cholesterol and risk of incident CVD. 

At each exam visit, participants were asked to provide a blood sample after ten or more hours of fasting. Subjects were considered to have T2DM if they reported using hypoglycemic medication or had blood glucose levels of 200 mg/dL or higher regardless of fasting status. Subjects with glucose levels of 126–199 mg/dL were also classified as having T2DM if they met one of the following criteria: (1) fasted for 10 h or more; (2) had a history of diabetes; or (3) were diagnosed with T2DM at the following exam without having gained 7% or more of body weight between exams. Classification of IFG at exams 1 and 2 was complicated due to uncertain fasting time; subjects not meeting the criteria for T2DM but who had a fasting glucose level ≥126 mg/dL at either of the first two exams was considered to have IFG. At all subsequent exams, subjects who fasted for 10 or more hours were considered to have IFG when their fasting glucose levels were between 100–125 mg/dL.

### 2.2. Dietary Assessment

Three-day diet records were collected at exam visit 3 (starting in 1984) and exam visit 5. Approximately 70% of participants completed diet records, which were then entered into the Nutrition Data System (NDS), a nutrient calculation program developed at the University of Minnesota, to calculate their daily intake of nutrients (i.e., energy, macronutrients, vitamins, and minerals). The usual intake of dietary cholesterol was estimated as the mean intake from up to six days of diet records.

### 2.3. Outcomes

Serum lipids explored in these analyses included LDL-cholesterol, HDL-cholesterol, the LDL:HDL ratio, and triglycerides. Fasting measures were taken at 4-year intervals from the baseline exam for these analyses (beginning at the time of dietary assessment) to the end of follow up. Standard lipid assessment methods were used as has been previously described [19].

CVD events in these analyses included fatal and non-fatal myocardial infarction, incident coronary heart disease diagnosis, and stroke. Incident CVD was determined by calculating person-years of follow up from the time of the final dietary cholesterol measurement to the first of the following events: occurrence of a CVD event, loss to follow up, date of death, or date of last exam (exam visit 8).

### 2.4. Potential Confounding Variables

Height and weight were measured at each exam visit. For the calculation of BMI (weight (kg)/[height (m)]^2^) at each exam visit, the exam-specific weight was divided by average adult height (with height censored at age 60 to eliminate effects of height loss). For example, height measurements for a 64-year-old subject at baseline (exam visit 3) were derived from measures at exam visits 1 and 2. Waist circumference was measured at exam visit 4. Physical activity was measured by questionnaire and a physical activity index was calculated as a weighted average of total hours of moderate and vigorous activity per day at exam visits 2 and 4. Smoking was assessed as the number of cigarettes smoked per day among current smokers and total pack-years of smoking was estimated by multiplying amount smoked by the number of years of smoking. Alcohol intake was also assessed at each exam visit by questionnaire. The potential confounders explored in these analyses included age, sex, BMI, waist circumference, education level, physical activity, smoking (cigarettes per day and total pack-years of smoking), energy intake, percentage of energy from carbohydrates, total fat, saturated fat and total protein, use of lipid-lowering medication, and alcohol intake. Only those factors that confounded the estimates by approximately 10% or more were included in the final models: age, pack-years of smoking, waist circumference, percentage of energy from carbohydrates and saturated fat, and use of lipid-lowering medication.

### 2.5. Statistical Analysis

To optimize study power, subjects were categorized into three sex-specific categories of dietary cholesterol intake, with the lowest tertile serving as the referent group. Repeated measures of lipid levels were compared across categories of dietary cholesterol intake using longitudinal mixed linear regression models, with unstructured covariance assumptions.

Prospective analyses were conducted using Cox proportional hazards models to examine the risk of cardiovascular disease associated with sex-specific tertiles of dietary cholesterol intake while adjusting for confounding by sex, age, smoking (pack-years), waist circumference, percentage of total energy from saturated fat and from carbohydrates, and use of lipid-lowering medications. All statistical analyses were performed with SAS (version 9.3; SAS Institute Inc., Cary, NC, USA).

## 3. Results

As shown in Table 1, dietary cholesterol intake among males was higher than among females in all three sex-specific tertiles of cholesterol intake. Cigarette smoking was highest among those in the highest tertile of cholesterol intake (20.9% vs. 16.0% pack-years in the highest and lowest tertiles). Lipid-lowering drug use was highest in the lowest tertile of intake (6.1%, 2.7%, and 1.5% in low, moderate, and high tertiles of intake, respectively).

**Figure 1:**
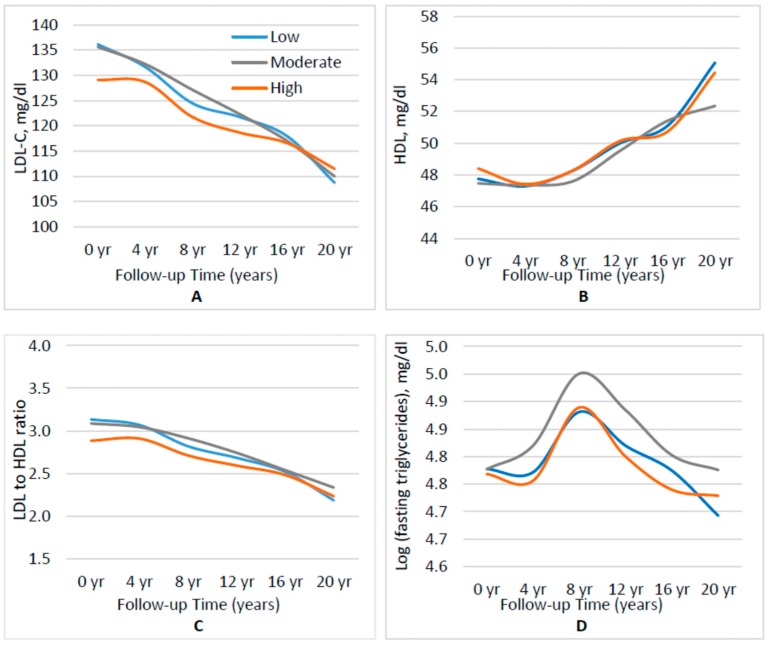
Plasma lipid levels according to sex-specific tertile (low, moderate, high) of dietary cholesterol intake. All analyses are adjusted for age, pack-years of cigarette smoking, waist circumference, use of lipid-lowering medications, and percentage of energy from carbohydrates and saturated fat. (**A**) fasting LDL cholesterol; (**B**) fasting HDL cholesterol; (**C**) LDL/HDL ratio; and (**D**) log of fasting triglycerides.

We began by examining the association between dietary cholesterol intakes and lipid levels over 20 years of follow up (Figure 1). After adjusting for sex, age, pack-years of smoking, waist circumference, percentage of energy from carbohydrates and saturated fat, and the use of lipid-lowering medication, there was no statistically significant association between dietary cholesterol intake and fasting LDL over time (*p* = 0.215 for highest vs. lowest tertile of intake) (Figure 1A). Similarly, there were no statistical differences in HDL-c (Figure 1B) or the LDL:HDL ratio (Figure 1C) across categories of dietary cholesterol intake (*p* = 0.4830 and *p* = 0.0678, respectively, for tertile 3 vs. tertile 1). Finally, Figure 1D shows that there was no consistent association between dietary cholesterol and fasting triglycerides over time (*p* = 0.8785 for tertile 3 vs. tertile 1).

**Figure 2:**
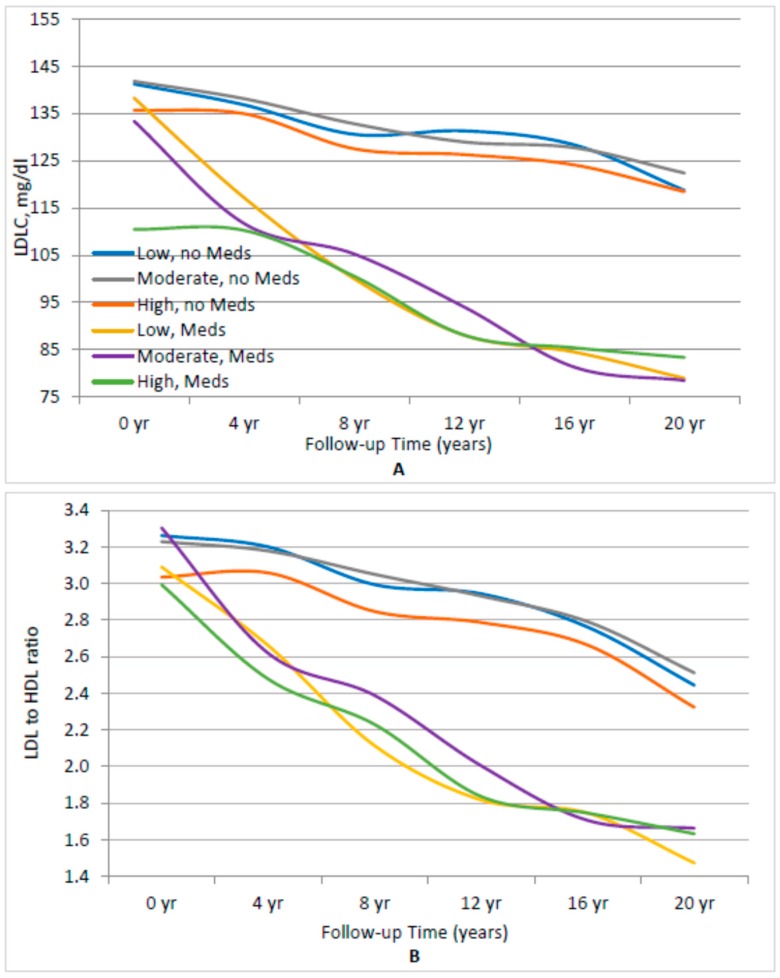
Association between sex-specific tertiles of cholesterol intake and LDL and the LDL/HDL ratio, stratifying by use of lipid-lowering medications. (**A**) Fasting LDL level. (**B**) LDL/HDL ratio. All analyses were adjusted for age, cigarette pack-years, waist circumference, lipid-lowering medicine, and percentage of energy from carbohydrates and saturated fats. Low, moderate, and high refer to the sex-specific tertiles of dietary cholesterol intake.

To further account for any impact of the use of lipid-lowering drugs among adults with prevalent IFG/T2DM, results were stratified in Figure 2 by medication use. Figure 2A finds no indication that subjects in the highest sex-specific tertile of dietary cholesterol intake had higher levels LDL levels, whether or not they were taking lipid-lowering medication. At each visit, it is evident that subjects currently taking lipid-lowering drugs had lower LDL levels than those not on lipid-lowering medications. However, in both cases, there was no association between dietary cholesterol intake and LDL (*p* = 0.1768 for highest vs. lowest dietary cholesterol intake among subjects not on lipid-lowering drugs; *p* = 0.2135 for highest vs. lowest dietary cholesterol intake among subjects taking lipid-lowering drugs).

In Figure 2B, those men and women with IFG or T2DM who were not taking lipid-lowering medications and who had higher intakes of dietary cholesterol had non-statistically significant lower LDL:HDL ratios (*p* = 0.0567). This was not the case for those individuals taking lipid-lowering drugs (*p* = 0.9849).

**Figure 3:**
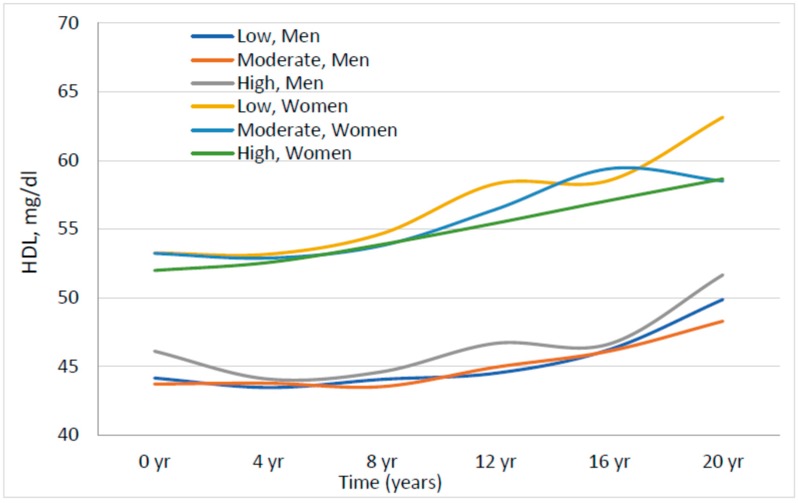
Association between sex-specific tertiles of dietary cholesterol and HDL, stratifying by sex. All analyses were adjusted for age, pack-years of cigarette smoking, waist circumference, lipid-lowering medicine, and percentage of energy from carbohydrates and saturated fats. Low, moderate, and high refer to the sex-specific tertiles of dietary cholesterol intake.

In Figure 3, we examined the association of dietary cholesterol intake with HDL among males and females separately. In all three sex-specific tertiles, HDL levels were lower in men than in women but there was no clear association between dietary cholesterol intake and HDL level in either men or women. 

For the analyses of dietary cholesterol intake and risk of CVD, we documented 210 incident CVD events (Table 2). For all subjects, those in the highest sex-specific tertile of dietary cholesterol had a 39% decreased risk of CVD compared with those in the lowest tertile of intake (HR: 0.61; 95% CI: 0.41, 0.90). In sex-stratified analyses, men in the highest sex-specific tertile of dietary cholesterol had a 43% lower risk of CVD, while women had a non-statistically significant 18% decreased risk compared with those in lowest tertiles of intake.

Finally, to evaluate whether lipid levels might be a causal intermediate in the relation between dietary cholesterol and CVD risk, we repeated these analyses after adjusting for LDL, HDL, and triglycerides in separate multivariable models (Supplemental Table S1). There was no change in the results.

## 4. Discussion

This study found no evidence of adverse association between dietary cholesterol and risk of CVD among adults with T2DM or prediabetes. In fact, after adjusting for confounding by other lifestyle factors, those with higher dietary cholesterol intakes had a lower long-term risk of developing CVD. These effects were stronger in men (who tended to have higher dietary cholesterol intakes) than women. Further, there was no adverse association between dietary cholesterol intake and changes in lipid levels (LDL, HDL, LDL:HDL ratio, or triglycerides) over 20 years of follow up in the Framingham Offspring Study. These results did not vary between those who were taking lipid-lowering medications and those who were not. Finally, there was no difference in the association between dietary cholesterol intake and HDL in men and women.

There are a limited number of previous studies that examine the effects of dietary cholesterol on cardiovascular risk among adults with IFG or T2DM [17,20]. Our results differ from those in the Health ABC Study in which 70–79-year-old subjects with T2DM were found to have a higher risk of CVD in association with higher dietary cholesterol intakes [17]. They are also at odds with results from the Nurses’ Health Study which also found that women with T2DM in the highest quintile of dietary cholesterol intake had higher CVD risk [20]. Both of these studies included generally healthy community-dwelling adults, while those selected for the current analyses were individuals with prevalent T2DM. It is possible that more health-conscious individuals with T2DM consumed fewer eggs and less meat and had other healthy behaviors. Thus, their lower risks of CVD may have resulted from a generally healthier lifestyle rather than their intakes of dietary cholesterol. In the Nurses’ Health Study analyses, the adverse association between dietary cholesterol and CVD risk was attenuated after controlling for confounding by other lifestyle and dietary factors. Still, residual confounding could further explain some of the apparent adverse effects of dietary cholesterol on CVD risk in these earlier studies.

Eggs are the primary source of dietary cholesterol in the American diet and some previous studies have used eggs as a surrogate measure for dietary cholesterol intake. Our results align with two previous meta-analyses of prospective cohort studies showing no association between dietary egg consumption and risk of CVD [16,21], and are at odds with other studies, including another meta-analysis of prospective studies and a separate large prospective cohort, suggesting that subjects with diabetes who consume more than one egg per day have a higher risk of developing CVD than those consuming less than one egg per day [14,15,22]. A large randomized controlled trial among individuals with IFG or T2DM found no elevation in LDL among those consuming 12 eggs per week compared with those who consumed 2 eggs per week over 3 months of follow up [23]. In addition, previous studies have shown that egg consumption is associated with increases in particle size for HDL and LDL, a finding that could explain the anti-atherogenic effect associated with dietary cholesterol intake [17]. 

An important strength of the current study is the availability of detailed dietary records, perhaps allowing for better control for potential confounding by other dietary factors. Thus, confounding by other dietary factors could explain some of the differences in the results between the current analyses and some earlier studies. Another strength of the current study includes its large sample size and long-term follow up (20 years after dietary cholesterol assessment). Finally, these results are strengthened by the inclusion of dietary cholesterol from all sources rather than from eggs alone, allowing for a more precise estimate of total dietary cholesterol effects on cardiovascular outcomes.

The 2015 *Dietary Guidelines* eliminated the previous long-standing restrictions on the intake of dietary cholesterol [7]. This change stemmed from the lack of evidence that dietary cholesterol was associated with risk of CVD among generally healthy adults. A question has remained about whether dietary cholesterol intake should be limited among individuals with prevalent diabetes out of concern for an excess risk of dyslipidemia or cardiovascular disease. This study addressed that important clinical question and found no evidence to support the need to restrict dietary cholesterol intake among adults with impaired fasting glucose or type 2 diabetes.

## 5. Conclusions

This analysis of prospective data identified no adverse association between dietary cholesterol and serum lipid levels or CVD risk amongst those with impaired fasting glucose or Type 2 diabetes. 

## Figures and Tables

**Table 1:** Baseline characteristics by sex-specific tertiles of dietary cholesterol intake.

Subject Characteristics	Sex-Specific Tertiles of Cholesterol Intake		
	Low (*N* = 330)	Moderate (*N* = 332)	High (*N* = 331)
Gender, Male (%)	198 (60.0)	199 (59.94)	198 (59.52)
Dietary Cholesterol, Male (mg)	71.0–225.0	225.1–324.5	324.6–1033.3
Dietary Cholesterol, Female (mg)	58.7–164.2	164.3–241.2	241.3–582.4
Age (years)	53.4 (7.2)	52.8 (7.9)	51.9 (7.4)
Cigarettes (pack-years)	16.0 (20.9)	17.9 (22.2)	20.9 (23.6)
Waist circumference (inches)	37.0(5.2)	37.5 (5.2)	38.4 (5.3)
Total energy intake (kcal)	1670 (436.3)	1930 (503.7)	2205 (530.8)
% of energy carbohydrate	209.7 (70.6)	220.7 (69.8)	234.3 (69.6)
% of energy saturated fat	19.0 (7.2)	25.6 (8.6)	33.1 (11.2)
% of energy protein	17.1 (3.6)	16.5 (3.1)	17.2 (3.1)
Physical activity (hrs/day)	12.7 (8.4)	12.8 (9.1)	13.3 (9.5)
Alcohol (grams/day)	11.6 (19.8)	12.9 (18.5)	14.0 (19.4)
Lipid-lowering drugs, *N* (%)	20 (6.1)	9 (2.7)	5 (1.5)

Values are mean (±SD) for continuous valuables; *N* and percentage for categorical variables.

**Table 2:** Risk of cardiovascular disease by category of intake of dietary cholesterol in Framingham Offspring Study adults.

Dietary Cholesterol (Sex-Specific Tertiles)	*N*	Person-Years	CVD Events	Hazard Ratio	95% CI
**ALL SUBJECTS**					
Low	298	4678.9	68	1.00	–
Moderate	305	4714.2	78	0.95	0.68–1.33
High	308	4941.1	64	0.61	0.41–0.90
**MEN**					
Low	172	2661.8	49	1.00	–
Moderate	180	2694.8	55	0.93	0.62–1.38
High	184	2896.3	46	0.57	0.36–0.91
**WOMEN**					
Low	126	2017.1	19	1.00	*Ref*
Moderate	125	2019.3	23	1.10	0.58–2.10
High	124	2044.8	18	0.82	0.38–1.73

Adjusted for age, sex (all subjects model), pack-years of smoking, waist circumference, percent of energy from saturated fat and carbohydrates, and use of lipid-lowering medication.

## Supplementary Materials

The following are available online at http://www.mdpi.com/2072-6643/10/6/770/s1, Table S1 Evaluating the intermediate effects of lipids on CVD risk among Framingham Offspring adults with Impaired Fasting Glucose or Type 2 Diabetes.
