# Supplementary material for: Dietary Cholesterol, Lipid Levels, and Cardiovascular Risk among Adults with Diabetes or Impaired Fasting Glucose in the Framingham Offspring Study
Source: Nutrients. 2018 Jun 14;10(6):770. doi: 10.3390/nu10060770 (PMC6024517; doi:10.3390/nu10060770)
Supplement: Supplementary file 1 [file nutrients-10-00770-s001.pdf]

Supplementary Table 1. Evaluating the intermediate effects of lipids on CVD risk among Framingham Offspring adults with Impaired Fasting Glucose or Type 2 Diabetes

	Model 1		Model 2		Model 3	
Dietary Cholesterol (Sex-specific tertiles)	HR	95% CI	HR	95% CI	HR	95% CI
**Low**	1.00	-	1.00	-	1.00	-
**Moderate**	0.95	0.68-1.32	0.93	0.67-1.31	0.95	0.68-1.33
**High**	0.62	0.41-0.91	0.62	0.42-0.91	0.61	0.41-0.90

Abbreviation: HR, hazard ratio. CI: confidence interval.

Model 1: Adjusted for age, sex, pack-years of smoking, waist circumference, percent of energy from carbohydrates and saturated fat, use of lipid-lowering medications, and LDL-cholesterol.

Model 2: : Adjusted for age, sex, pack-years of smoking, waist circumference, percent of energy from carbohydrates and saturated fat, use of lipid-lowering medications, and log of triglycerides.

Model 3: Adjusted for age, sex, pack-years of smoking, waist circumference, percent of energy from carbohydrates and saturated fat, use of lipid-lowering medications, and HDL-cholesterol.
